# Impact of Molecular Modification on the Efficiency of Recombinant Baculovirus Vector Invasion to Mammalian Cells and Its Immunogenicity in Mice

**DOI:** 10.3390/v14010140

**Published:** 2022-01-13

**Authors:** Hao Zheng, Yong Pan, Xiong Wang, Weibin Tian, Lunguang Yao, Jingchen Sun

**Affiliations:** 1 Guangdong Provincial Key Laboratory of Agro-Animal Genomics and Molecular Breeding & Subtropical Sericulture and Mulberry Resources Protection and Safety Engineering Research Center, College of Animal Science, South China Agricultural University, Guangzhou 510642, China; zhenghao_scau@163.com (H.Z.); panyongscau@gmail.com (Y.P.); wangttx@outlook.com (X.W.); tian46102256@163.com (W.T.); 2 Henan Provincial Engineering and Technology Center of Health Products for Livestock and Poultry, College of Life Science and Agricultural Engineering, Nanyang Normal University, Nanyang 473061, China

**Keywords:** baculovirus display system, envelope protein, high-efficiency display, multiple vaccine

## Abstract

The baculovirus display system (BDS), an excellent eukaryotic surface display technology that offers the advantages of safety, efficiency, and economy, is widely used in biomedicine. A previous study using rBacmid-Δgp64-ires-gp64 expressed in low copy numbers of the *gp64* gene achieved high-efficiency expression and co-display of three fluorescent proteins (GFP, YFP, and mCherry). However, low expression of GP64 in recombinant baculoviruses also reduces the efficiency of recombinant baculovirus transduction into mammalian cells. In addition, the baculovirus promoter has no expression activity in mammalian cells and thus cannot meet the application requirements of baculoviral vectors for the BDS. Based on previous research, this study first determined the expression activity of promoters in insect *Spodoptera frugiperda* 9 cells and mammalian cells and successfully screened the very early promoter *pie1* to mediate the co-expression of multiple genes. Second, utilizing the envelope display effect of the INVASIN and VSVG proteins, the efficiency of transduction of recombinant baculovirus particles into non-host cells was significantly improved. Finally, based on the above improvement, a recombinant baculovirus vector displaying four antigen proteins with high efficiency was constructed. Compared with traditional BDSs, the rBacmid-Δgp64 system exhibited increased display efficiency of the target protein by approximately 3-fold and induced an approximately 4-fold increase in the titer of serum antibodies to target antigens in Bal B/c mice. This study systematically explored the application of a new multi-gene co-display technology applicable to multi-vaccine research, and the results provide a foundation for the development of novel BDS technologies.

## 1. Introduction

Insect baculoviruses are double-stranded circular DNA viruses with a typical envelope structure in nature [1]. The typical characteristic of insect baculoviruses is that the virus particle has two different structural forms, namely BV (budded virus) and ODV (occlusion-derived virus) [2]. BVs and ODVs are responsible for cell and oral infections, respectively, and they are widely used as high-efficiency expression vectors and biological pesticides because of their high safety, environmental friendliness, and simplicity of genetic manipulation [3,4,5]. Baculoviral vectors have been widely used in the fields of antigen presentation, gene therapy, and DNA vaccines [6,7]. Based on the baculovirus display system (BDS), a target protein is displayed on the surface of a baculovirus capsule and cell membrane with high efficiency and activity using a co-expression technology [8]. The classic display technology involves inserting the target gene between the signal peptide and the extracellular domain of the envelope protein gene, which can display the target stably and efficiently without affecting the life cycle of the baculovirus [9,10]. Proteins widely used for display include low pH-mediated membrane-promoting fusion proteins, including GP64 (group I type baculovirus), F protein (group II type baculovirus), and non-polar protein VSVG (vesicular stomatitis virus G protein) [11,12,13].

When a baculovirus infects a host cell, the virus binds to the cell surface in a non-specific manner to complete the mutual recognition of receptors and then enters the cell via endocytosis, pinocytosis, or membrane fusion [14]. Studies have found that dynein and clathrin can mediate the endocytosis of membrane proteins under low-pH conditions, which in turn triggers fusion between the capsule membrane and the cell membrane [15]. Furthermore, protein inhibitors can significantly reduce the invasion efficiency of pseudo-type recombinant viruses, indicating that receptor recognition involves BV particle envelope proteins (such as GP64) [15]. Further studies have shown that a potential cholesterol recognition amino acid sequence is located in the specific recognition region of the GP64 protein [16]. Moreover, membrane phospholipids and heparan sulfate proteoglycan sites also mediate the invasion process of BV particles [17].

To date, many envelope proteins in the BDS system have been studied, including VSVG, INVASIN (Invasins protein), and influenza A virus H protein [18]. A feature common to these proteins is that they play a role in clathrin-dependent endocytosis in the invasion processes of viruses and bacteria [19]. VSVG is a non-polar protein on the surface of the vesicular stomatitis virus envelope and directs the process of vesicular stomatitis virus invasion of host cells [20]. Vesicular stomatitis virus can invade cells in a wide range of hosts, including mammals, birds, fish, and insects. VSVG is often used as an immune enhancement factor because it induces immune responses and neutralizes specific antibodies. Functionally, VSVG is similar to GP64, which exhibits changes in the spatial conformation of the protein under low-pH conditions (pH < 6.1) [20]. The INVASIN protein is encoded by the *inv* gene of *Yersinia pseudotuberculosis* and binds to the *β1* chain of integrin to specifically recognize *αβ1* family proteins [21]. In previous research, we elucidated the process of INVASIN-mediated direct invasion of *Spodoptera frugiperda* 9 (Sf9) and BmN cells by *Escherichia coli* Sw106 [8]. Therefore, we speculated that INVASIN may also increase the efficiency and range of baculovirus infection. In addition, INVASIN is a mature immune adjuvant that can significantly increase the titer of specific antibodies.

In previous studies, we achieved the efficient display of three fluorescent proteins on the surface of the baculovirus capsule and insect cell membrane through the low expression of GP64 mediated by deletion of the gp64 and internal ribosome entry site (ires) genes in Bacmid. However, the baculoviral promoter cannot be expressed efficiently in mammalian cells, and low expression of GP64 also reduces the invasion efficiency of recombinant baculoviruses [8]. In this study, we conducted a screening for promoters that can be efficiently expressed in mammalian and insect cells in combination with surface display of non-homologous envelope proteins (INVASIN and VSVG) in order to construct a recombinant baculovirus particle with high display and transduction efficiency. In addition, the immunogenicity of the co-displayed antigen was explored in mice. The results of this research could lead to an improved BDS and lay an experimental foundation for the development of insect baculovirus vectors for multiple vaccines.

## 2. Materials and Methods

### 2.1. Bacterial Strains, Plasmids, Viral Bacmid, Reagents, Cells, and Mice

*Escherichia coli* strains DH10B, BW23474, and TOP10 were used for the propagation of Bacmid, *R6kγ* origin-derived plasmids, and *ColE1* origin-derived plasmids, respectively. *Escherichia coli* Sw106 Bacmids containing baculoviral plasmid, pHelper, and pGB_2_Ωinv were constructed as described previously [22,23]. The plasmids pUCDM and pFBDM were obtained from Prof. Richmond. pFBDM and pUCDM as well as a modified Bacmid encoding gentamycin and chloramphenicol resistance genes by mini-Tn7 and Cre-loxp transposition were developed in our previous studies [24,25]. pFBDM-p64-gfp-ires-gp64 containing the 59-UTR ires sequence was constructed in a previous study [26]. The *Taq*, restriction enzymes, and T_4_ DNA ligase were purchased from NEB (New England Biolabs, Ipswich, MA, USA), whereas DL-α-ε diaminopimelic acid was obtained from Sigma (cat. D1377, St. Louis, MI, USA). Low-salt medium (10 g of tryptone, 5 g of NaCl, and 5 g of yeast extract in 1 L of broth (pH 7.5)) was used for cloning and growing plasmids encoding resistance genes. Dual-luciferase reporter assay system was purchased from Promega (cat. E1910, Madison, WI, USA), and the instructions can be obtained online (www.promega.com/protocols, accessed on 16 June 2021). Enzyme-linked immunosorbent assay (ELISA) virus antigens and serum antibody kits were purchased from *Ziker* (Shenzhen, China) biological (www.zikerbio.com, accessed on 25 August 2021), including PCV2-orf2 (cat. p6624/p6591), CSFV-E2 (cat. p9731/p6609), and PRRSV-GP5 (cat. p6455/p9732). Sf9 cells were maintained at 27 °C in serum-free SF-900 II medium (Thermo Fisher Scientific, Waltham, MA, USA). Baby hamster kidney 21 (BHK-21, cat. CL-0034) and 293T (cat. CL-0005) cells were purchased from Procell (Wuhan, China) and cultured in Dulbecco’s Modified Eagle Medium containing 10% fetal bovine serum (FBS) at 37 °C in 5% CO_2_. SPF BALB/c inbred mice were purchased from the Experimental Animal Center of Guangzhou University of Traditional Chinese Medicine (license no. SCXK-yue-2013-0034).

### 2.2. Construction of Donor Vectors

**Figure 1:**
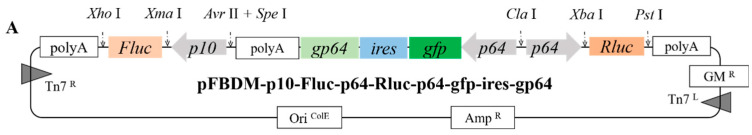

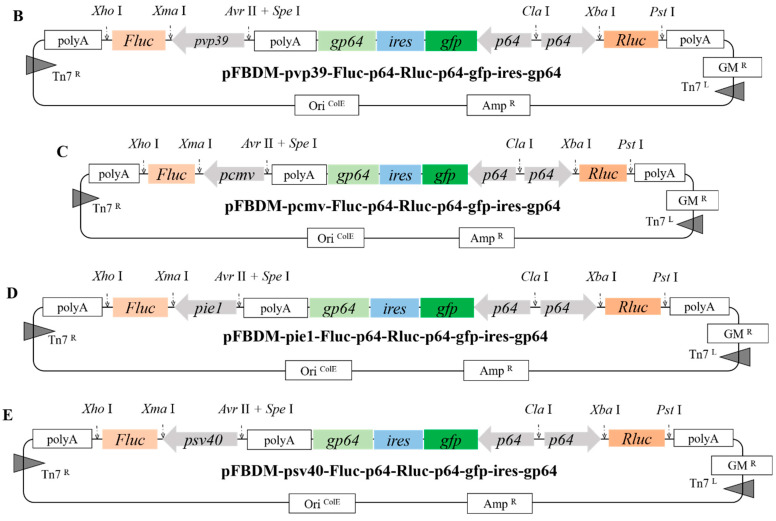
Construction of recombinant plasmid pFBDM carrying different promoters (*p10*, *pvp39*, *pcmv*, *psv40*, and *pie1*). The gene expression cassette p64-Rluc-polyA was used for the expression of the *Renilla* luciferase (as reference). The cassette p64-gfp-ires-gp64-polyA could express GP64 in low-expression as compensation for *gp64*-deficient Bacmid. Different promoters in the cassette pX-Fluc-polyA (pX: *p10*, *pvp39*, *pcmv*, *pie1*, and *psv40*) mediate the expression of *Firefly* luciferase. (**A**–**E**) The map of plasmids pFBDM-pX-Fluc-p64-Rluc-p64-gfp-ires-gp64 (pX: *p10* (**A**), *pvp39* (**B**), *pcmv* (**C**), *pie1* (**D**), and *psv40* (**E**)).

To screen for promoters with higher activity in insect and mammalian cells, the recombinant plasmid pFBDM-pX-Fluc-p64-Rluc-p64-gfp-ires-gp64 (pX: *p10*, *pvp39*, *pcmv*, *pie1,* and *psv40*) was constructed (Figure 1). *p10* and *pvp39* are late and early-late baculovirus promoters, respectively. The promoters *pcmv* (Cytomegalovirus), *pie1* (white shrimp syndrome virus), and *psv40* (Simian vacuolating virus 40) exhibit high activity and are widely used in mammalian cells. The *Renilla* luciferase gene (*Rluc*, www.promega.com, accessed on 21 September 2021) was cloned using the primers *Rluc*-F and *Rluc*-R (Table 1) and inserted into the plasmid via the *Xba*I and *Pst*I restriction sites (Figure 1). Similarly, the firefly luciferase gene (*Fluc*, www.promega.com, accessed on 21 September 2021) was cloned using the *Fluc*-F and *Fluc*-R (Table 1) primers and then ligated into the plasmid via the *Xho*I and *Xma*I restriction sites (Figure 1). A total of five promoters (*p10*, *pvp39*, *pcmv*, *pie1*, and *psv40*) were amplified by PCR using the corresponding primers listed in Table 1, all of which were ligated into the plasmid via the *Spe*I and *Xma*I restriction sites (Figure 1). As a *gp64*-deficient Bacmid was used in this study, *ires*-mediated low expression of GP64 (*ires-gp64* expression cassette) was required. pFBDM-p64-gfp-ires-gp64 was digested using *Cla*I and *Avr*II to release the p64-gfp-ires-gp64-polyAsv40 DNA [8]. The fragment was then cloned using the *Spe*I and *Cla*I sites to obtain the recombinant donor pFBDM-pX-Fluc-p64-Rluc-p64-gfp-ires-gp64 (pX: *p10*, *pvp39*, *pcmv*, *pie1*, and *psv40*).

**Figure 2:**
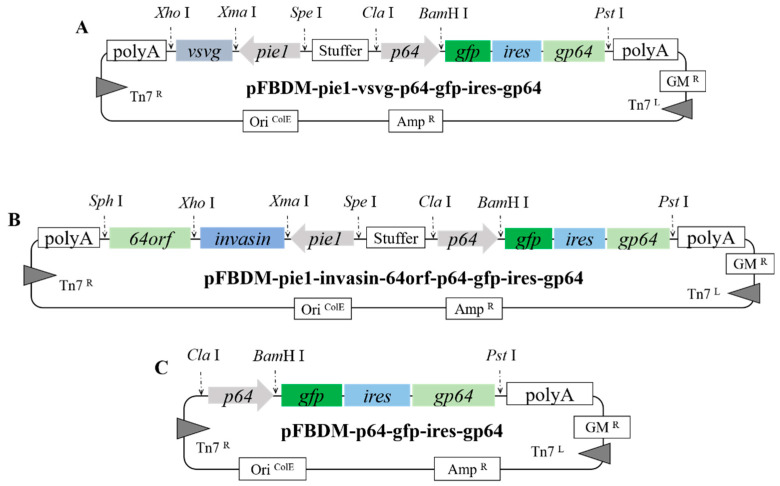
Construction of recombinant plasmids pFBDM carrying *vsvg* and *invasin*. The gene expression cassettes pie1-vsvg-polyA and pie1-invasin-64orf-polyA realize the modification of the recombinant baculovirus envelope through protein fusion, respectively, to improve the efficiency of recombinant baculovirus transduction into mammalian cells. (**A**) The map of the plasmid pFBDM-pie1-vsvg-p64-gfp-ires-gp64. (**B**) The map of the plasmid pFBDM-pie1-invasin-64orf-p64-gfp-ires-gp64. (**C**) The map of the plasmid pFBDM-p64-gfp-ires-gp64 (as control).

To determine the invasion efficiency of AcBV-Δgp64-ires-gp64 in insect and mammalian cells, the *vsvg* and *invasin* genes were fused to the recombinant plasmid pFBDM-p64-gfp-ires-gp64. The promoter *pie1* fused with the *gp64* signal peptide was cloned using the primers *pie1*-F and *pie1*-R shown in Table 1 and introduced into the recombinant plasmid pFBDM-ie1-p64-gfp-ires-gp64 via the *Spe*I and *Xma*I restriction sites. The envelope gene *vsvg* was amplified using the primers *vsvg*-F and *vsvg*-R and integrated downstream of *pie1* via the *Xma*I and *Xho*I restriction sites to obtain the recombinant plasmid pFBDM-pie1-vsvg-p64-gfp-ires-gp64 (Figure 2). The *invasin* gene, lacking a stop codon, was amplified using the primers *invasin*-F and *invasin*-R (Table 1) and then ligated into the plasmid via the *Xma*I and *Xho*I restriction sites. The fused *gp64* gene (*64orf*, amplified using the primers *64ORF*-F and *64ORF*-R) was inserted via the *Xho*I and *Sph*I restriction sites to generate the recombinant plasmid pFBDM-pie1-invasin-64orf-p64-gfp-ires-gp64 (Figure 2).

**Figure 3:**
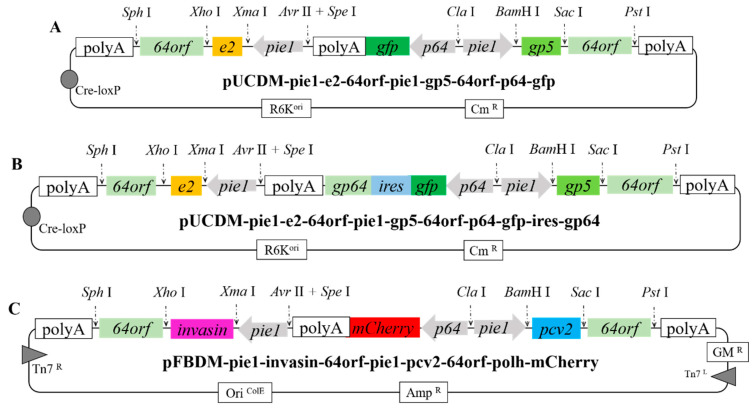
Construction of recombinant plasmids carrying antigen fusion genes. Four target genes (*e2*, *gp5*, *pcv2,* and *invasin*) were, respectively, fused with the membrane gene *64orf* to realize the display of the fused protein on the surface of the recombinant baculovirus envelope. (**A**) The map of plasmid pUCDM-pie1-e2-64orf-pie1-gp5-64orf-p64-gfp. (**B**) The map of plasmid pUCDM-pie1-e2-64orf-pie1-gp5-64orf-p64-gfp-ires-gp64. (**C**) The map of plasmid pFBDM-pie1-invasin-64orf-pie1-pcv2-64orf-polh-mCherry, respectively.

Similarly, four antigen genes (*pcv2* (orf 2, porcine circovirus type 2), *e2* (classical swine fever virus), *gp5* (porcine reproductive and respiratory syndrome virus), and *invasin* (*Yersinia pseudotuberculosis*)), which the stop codons, were deleted to construct a multiple vaccine based on rBacmid-Δgp64-ires-gp64. The fused genes e2-64orf (*E64*), gp5-64orf (*G64*), pcv2-64orf (*P64*), and invasin-64orf (*I64*) were inserted into the plasmid via restriction sites to generate the multi-gene recombinant vectors pUCDM-pie1-e2-64orf-pie1-gp5-64orf-p64-gfp, pUCDM-pie1-e2-64orf-pie1-gp5-64orf-p64-gfp-ires-gp64, and pFBDM-pie1-invasin-64orf-pie1-pcv2-64orf-p64-mCherry (Figure 3). In addition, introduction of the fluorescence-producing genes *gfp* and *mCherry* enabled direct observation of infection of cells with recombinant baculoviruses.

### 2.3. Introduction of Foreign Genes into rBacmid

The foreign gene carried by pFBDM was recombined into the Bacmid by mini-Tn7 transposition (rBacmid). rBacmid-Δgp64-pX-Fluc-p64-Rluc-p64-gfp-ires-gp64 (pX: p10, pvp39, pcmv, pie1, and psv40), rBacmid-Δgp64-p64-gfp-ires-gp64, rBacmid-Δgp64-ie1-vsvg-p64-gfp-ires-gp64, and rBacmid-Δgp64-pie1-invasin-64orf-p64-gfp-ires-gp64 were successfully obtained through antibiotic screening, blue and white spot screening, and PCR identification, respectively.

The fusion genes *E64*, *G64*, and *gfp* carried by the plasmid pUCDM were introduced into the Bacmid via Cre-loxP-specific transposition. Blue/white spot and antibiotic screening were used to obtain *E. coli* Sw106 rBacmid-pie1-e2-64orf-pie1-gp5-64orf-polh-gfp and *E. coli* Sw106 rBacmid-Δgp64-pie1-e2-64orf-pie1-gp5-64orf-polh-gfp-ires-gp64. Furthermore, pcv2-64orf (*P64*) and invasin-64orf (*I64*) carried by the plasmid pFBDM-pie1-invasin-64orf-pie1-pcv2-64orf-polh-mCherry were introduced into the rBacmid via mini-Tn7 transposition. Two-step transposition resulted in the generation of rBacmid containing four antigen fusion genes (*E64*, *G64*, *P64*, and *I64*) and two fluorescent genes (*gfp* and *mCherry*), which were designated rBacmid-Δgp64-E64-G64-I64-P64-GM-Igp64 and rBacmid-E64-G64-I64-P64-GM-Igp64, respectively [8].

### 2.4. Production of Recombinant Baculovirus

*Escherichia coli* Sw106 cells harboring the rBacmid encoding foreign genes were cultured until the optical density at 600 nm reached 0.5–1. The bacteria were collected by centrifugation (3000× *g*) and resuspended in serum-free insect medium. Aliquots of the bacterial suspension were adjusted to different densities (10^5^–10^8^ cells/mL) using serum-free Grace’s insect medium [22]. Next, Sf9 cells were cultured overnight in a 12-well plate until the cell density was approximately 70–80%. The supernatant was discarded, and different concentrations of bacteria were added to the corresponding wells. After culturing at 28 °C for 4–5 h, the bacteria in each well were washed out using serum-free Grace’s insect medium, and then, 500 μL of fresh insect medium (with 10% FBS and 0.075% penicillin) was added and incubated for 4–5 days post infection (dpi). Infected Sf9 cells were identified by examining the fluorescence in the corresponding wells under a fluorescence microscope (Eclipse Ti-S, Nikon, Tokyo, Japan). In order to obtain a stable titer of recombinant virus, the supernatant was collected and used to infect Sf9 cells again. Fluorescence appeared again at 3–4 dpi, indicating that the recombinant baculovirus was successfully constructed and distributed in the cell supernatant.

### 2.5. Determination of Recombinant Baculovirus Titer

Early exponential phase Sf9 cells were diluted to 10^6^ cells/mL with SF-900 II serum-free medium, and then, a 100-µL working volume was placed into different micro-wells in a 96-well plate. Serial 10-fold baculovirus dilutions were also prepared to 10^−8^ using SF-900 II insect medium. The cell culture medium was removed, and 100 µL of each virus dilution was added (12 wells per dilution) to the cell monolayer and then incubated at 28 °C. Following 4 h of incubation, the supernatant in each well was replaced with 100 µL of fresh insect medium. Plates were checked once daily for 4–5 days until the fluorescence was observed to reach a maximum. The baculovirus titer is expressed as the 50% tissue culture infective dose according to the standard method of Reed and Muench.

### 2.6. Baculovirus Purification and Transmission Electron Microscopy (TEM)

The supernatant of Sf9 cells infected with recombinant baculovirus (multiplicity of infection (MOI) = 1) was collected at 4 dpi, and baculoviruses in the supernatant were purified by two rounds of sucrose gradient ultracentrifugation according to standard methods. The purified baculoviruses were adsorbed onto glow discharge-activated carbon-coated grids for 2 min. The sample-coated grids were washed three times with distilled water, following by negative staining with 1% uranyl acetate for 45 s. Images were acquired using an Talos F200S transmission electron microscope (FEI, Hillsboro, OR, USA).

### 2.7. Dual-Glo Luciferase Assay System

Insect cells or mammalian cells were cultured overnight in a 12-well plate and expanded to the density of 80–90%. Recombinant baculovirus was directly added at a MOI of 1.0 and incubated for 6–8 h, after which the medium was replaced with fresh cell culture medium. The cells were then cultured for 48–96 h to allow for infection, and the cell infection rate was determined based on fluorescence. The cells were pelleted and washed with 1× PBS, and evaluation of *Renilla* and *Firefly* luciferase expression was determined according to the manufacturer’s instructions.

### 2.8. ELISA

A standard curve of absorbance versus concentration of antibody/antigen standard was prepared for each ELISA. According to the assay instructions, diluted sample (mouse serum and purified recombinant baculovirus) was fully mixed with HRP-conjugated reagent and incubated in the dark at 37 °C for 60 min. The sample was then mixed with chromogen solution and stop solution for 10 min, and the optical density at 400 nm was determined for each well.

### 2.9. Statistical Analysis

All values are presented as the mean ± standard deviation. GraphPad Prism 7 software (GraphPad Software, San Diego, CA, USA) was used for data analyses. One-way analysis of variance followed by Tukey’s post-hoc test was used to determine significant difference. *p* < 0.05 was considered statistically significant.

## 3. Results

### 3.1. Determination of Promoter Expression in Insect and Mammalian Cells

**Figure 4:**
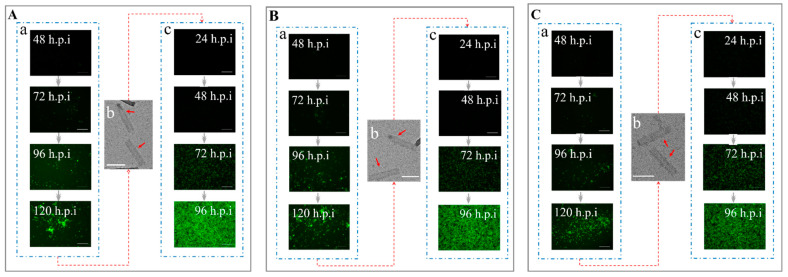

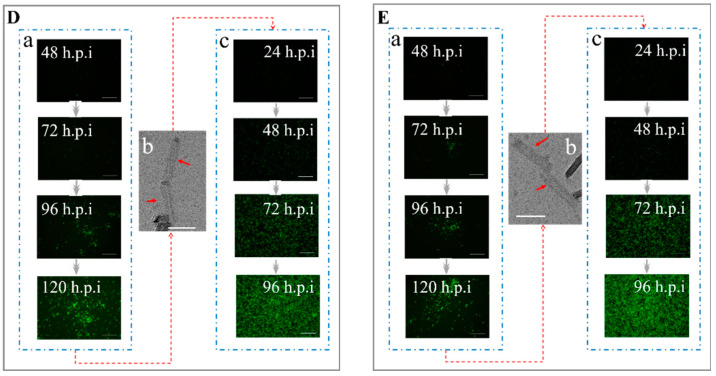
Construction of AcBVs with firefly luciferase expression mediated by five different promoters. (**A**) Construction of recombinant AcBV-Δgp64-p10-Fluc-p64-Rluc-p64-gfp-ires-gp64. (**a**) The recombinant rBacmid-Δgp64-p10-Fluc-p64-Rluc-p64-gfp-ires-gp64 was used to invade Sf9 cells (48–120 h, Bar = 200 µm). (**b**) Supernatant of Sf9 cells was collected and purified by centrifugation, and the progeny AcBV-Δgp64-p10-Fluc-p64-Rluc-p64-gfp-ires-gp64 was observed by TEM (Bar = 200 nm). (**c**) The AcBV-Δgp64-p10-Fluc-p64-Rluc-p64-gfp-ires-gp64 in the supernatant was used to re-infect Sf9 cells to complete the replication of the baculovirus (24–96 h, Bar = 200 µm). Similarly, (**B**–**E**) indicates construction of recombinant baculovirus AcBV-Δgp64-pvp39-Fluc-p64-Rluc-p64-gfp-ires-gp64 (**B**), AcBV-Δgp64-pcmv-Fluc-p64-Rluc-p64-gfp-ires-gp64 (**C**), AcBV-Δgp64-pie1-Fluc-p64-Rluc-p64-gfp-ires-gp64 (**D**), and AcBV-Δgp64-psv40-Fluc-p64-Rluc-p64-gfp-ires-gp64 (**E**), respectively.

The Sf9 cells infected with rBacmid-Δgp64-pX-Fluc-p64-Rluc-p64-gfp-ires-gp64 (pX: *p10*, *pvp39*, *pcmv*, *pie1,* and *psv40*) turned green (Figure 4). Within 48 to 120 h post infection (h.p.i), the number of green cells gradually increased, and the level of fluorescence reached a maximum at 120 h.p.i (Figure 4), indicating that the baculoviruses were propagating. The culture supernatant was collected and centrifuged at 80,000× *g*, and the cell pellet was observed by TEM, which indicated that the recombinant baculovirus particles AcBV-Δgp64-pX-Fluc-p64-Rluc-p64-gfp-ires-gp64 (pX: *p10*, *pvp39*, *pcmv*, *pie1,* and *psv40*) were constructed successfully (Figure 4). When the viruses were collected and used to re-infect Sf9 cells (MOI = 1), green cells were observed at 24 h.p.i (Figure 4). Similar to infection with the first-generation strain, fluorescence gradually increased within 24–96 h.p.i and reached a maximum at 96 h.p.i (Figure 4).

**Figure 5:**
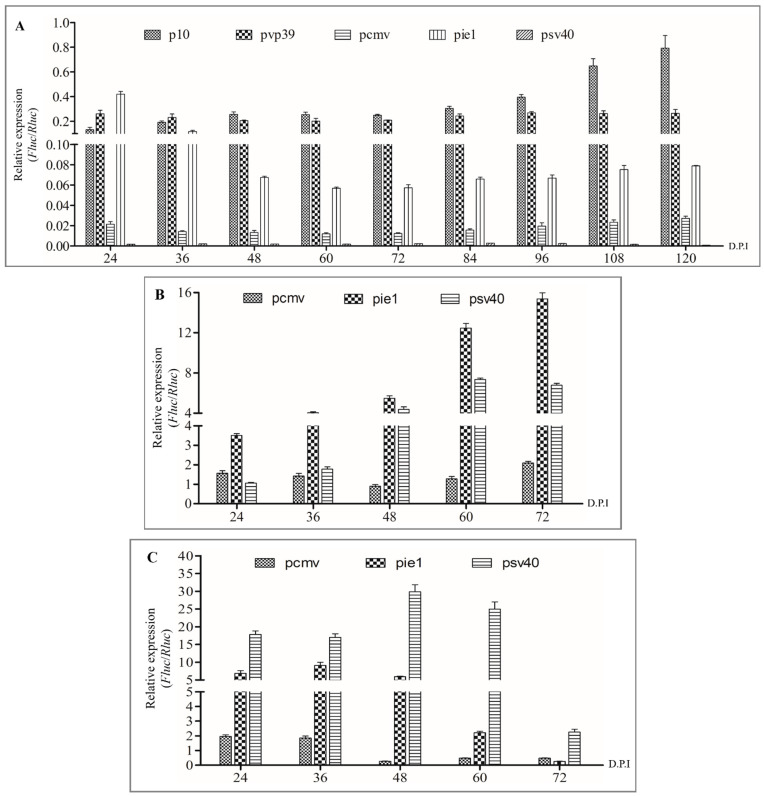
Determination of promoter expression in Sf9 and mammalian cells. (**A**) Promoters mediate the relative expression of *Firefly* luciferase (*Fluc*) compared with *Renilla* luciferase (*Rluc*) in Sf9 cells (*Fluc*/*Rluc*). (**B**) Mammalian promoters mediate the relative expression of *Fluc*/*Rluc* in 293T cells. (**C**) Mammalian promoters mediate the expression of *Fluc*/*Rluc* in BHK-21 cells.

The dual-luciferase reporter results showed that the promoters *pie1*, *p10*, and *pvp39* exhibited different expression timing and high expression activity in Sf9 cells compared with the *p64* promoter in the early stage. The expression efficiency of *pie1* was the highest in the early stage (24 h) of infection but decreased rapidly with the continuation of infection (Figure 5A). In addition, *pcmv* and *psv40* also mediated the expression of target genes in Sf9 cells, but the expression efficiency was insufficient (Figure 5A). Within 72 h of recombinant baculovirus invasion of 293T and BHK-21 cells, three mammalian cell promoters successfully expressed active luciferase. In particular, *pie1* and *psv40* exhibited higher early expression efficiency and longer expression time (Figure 5B,C). Interestingly, the expression rate of *pie1* in BHK-21 cells initially increased and then decreased, with maximum expression efficiency occurring within 36 h of infection (Figure 5C). Furthermore, the expression efficiency of the *pie1* promoter was better than that of *pcmv* and *psv40*, thus meeting the BDS requirements for high display efficiency and cross-host expression of foreign genes.

### 3.2. The Efficiency of Transducing Mammalian Cells by BV with Envelope Modification

**Figure 6:**
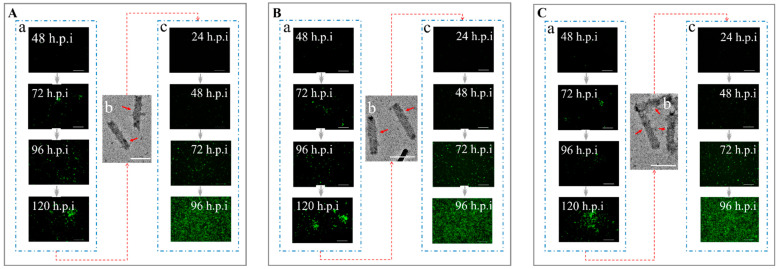
Construction of AcBVs carrying *vsvg* and *invasin*. (**A**) Construction of recombinant AcBV-Δgp64-pie1-vsvg-p64-gfp-ires-gp64. (**a**) The recombinant rBacmid-Δgp64-pie1-vsvg-p64-gfp-ires-gp64 was used to invade Sf9 cells (48–120 h, Bar = 200 µm). (**b**) Supernatant of Sf9 cells was collected and purified by centrifugation, and the progeny AcBV-Δgp64-pie1-vsvg-p64-gfp-ires-gp64 was observed by TEM (Bar = 200 nm). (**c**) The AcBV-Δgp64-pie1-vsvg-p64-gfp-ires-gp64 in the supernatant was used to re-infect Sf9 cells to complete the replication of the baculovirus (24–96 h, Bar = 200 µm). Similarly, (**B**,**C**) indicates construction of recombinant baculovirus AcBV-Δgp64-pie1-invasin-64orf-p64-gfp-ires-gp64 (**B**) and AcBV-Δgp64-p64-gfp-ires-gp64 ((**C**), as control), respectively.

Using liposome-free transfection and Bac-to-Bac technology, rBacmid-Δgp64-pie1-vsvg-p64-gfp-ires-gp64, rBacmid-Δgp64-pie1-invasin-64orf-p64-gfp-ires-gp64, and rBacmid-Δgp64-p64-gfp-ires-gp64 carrying foreign genes were used to infect Sf9 cells. Green fluorescence increased continuously over 48–120 h, indicating that *E. coli* Sw106 invaded and the target genes were expressed successfully (Figure 6). After the recombinant baculovirus in the supernatant was concentrated and purified, the mature baculovirus particles were clearly observed by TEM (Figure 6). The recombinant baculoviruses were collected and used to re-infect normal Sf9 cells to obtain stable high-virulence particles (Figure 6), which indicated that the recombinant baculovirus AcBV-Δgp64-pie1-vsvg-p64-gfp-ires-gp64 (virus titer 2.95 × 10^7^ PFU), AcBV-Δgp64-pie1-invasin-64orf-p64-gfp-ires-gp64 (virus titer 3.46 × 10^7^ PFU), and AcBV-Δgp64-p64-gfp-ires-gp64 (virus titer 8.69 × 10^7^ PFU) were constructed successfully.

**Figure 7:**
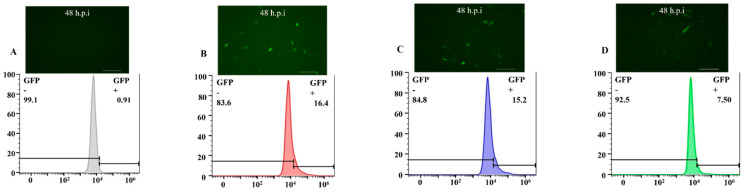
Efficiency of transducing BHK-21 cells by AcBV with envelope modification. (**A**) Measure the number of BHK-21 cells expressing green fluorescence by flow cytometry. (**B**) Measure the number of BHK-21 cells expressing green fluorescence by flow cytometry, which the AcBV-Δgp64-pie1-invasin-64orf-p64-gfp-ires-gp64 infected for 48 h. (**C**) Measure the number of BHK-21 cells expressing green fluorescence by flow cytometry, which the AcBV-Δgp64-pie1-vsvg-p64-gfp-ires-gp64 infected for 48 h. (**D**) Measure the number of BHK-21 cells expressing green fluorescence by flow cytometry, which the AcBV-Δgp64-p64-gfp-ires-gp64 infected for 48 h (as control).

Baculovirus samples were adjusted to the same titer (virus titer 1 × 10^7^ PFU) and then incubated with BHK-21 and 293T cells for 4–6 h, then the supernatant was replaced with DMEM medium and incubated at 28 °C for 48 h. The green fluorescence in BHK-21 and 293T cells indicated that the recombinant virus invaded these mammalian cells and expressed GFP successfully (Figure 7). Enumeration of fluorescent cells by flow cytometry showed that the transduction efficiency of the recombinant baculovirus with INVASIN and VSVG displayed on BHK-21 cells increased to 16.4% and 15.2%, respectively, compared with the control (*p* < 0.05) (Figure 7). 

**Figure 8:**
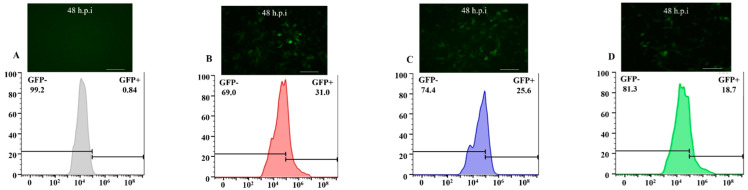
Efficiency of transducing 293T cells by AcBV with envelope modification. (**A**) Measure the number of 293T cells expressing green fluorescence by flow cytometry. (**B**) Measure the number of 293T cells expressing green fluorescence by flow cytometry, which the AcBV-Δgp64-pie1-invasin-64orf-p64-gfp-ires-gp64 infected for 48 h. (**C**) Measure the number of 293T cells expressing green fluorescence by flow cytometry, which the AcBV-Δgp64-pie1-vsvg-p64-gfp-ires-gp64 infected for 48 h. (**D**) Measure the number of 293T cells expressing green fluorescence by flow cytometry, which the AcBV-Δgp64-p64-gfp-ires-gp64 infected for 48 h (as control).

Similarly, the transduction efficiency of baculoviruses carrying INVASIN and VSVG to 293T cells increased to 31.0% and 25.6%, respectively, compared with the control (*p* < 0.05) (Figure 8). To sum up, the results indicated that envelope display of INVASIN and VSVG could increase the visual invasion rate in non-host cells, significantly.

### 3.3. Determination of the Co-Display Efficiency of Multiple Bacmid-Δgp64 Genes

**Figure 9:**
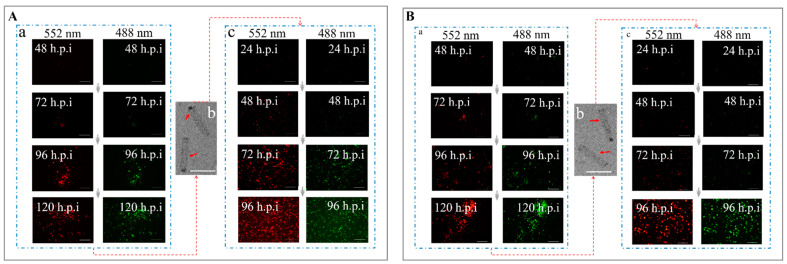
Construction of recombinant baculoviruses AcBV-E64-G64-I64-P64-G-M and AcBV-Δgp64-E64-G64-I64-P64-GM-Igp64. (**A**) Construction of recombinant AcBV-E64-G64-I64-P64-G-M. (**a**) The recombinant rBacmid-E64-G64-I64-P64-G-M-Igp64 was used to invade Sf9 cells (48–120 h, Bar = 200 µm, Excitation = 488 nm and 522 nm). (**b**) Supernatant of Sf9 cells was collected and purified by centrifugation, and the progeny AcBV-E64-G64-I64-P64-G-M-Igp64 was observed by TEM (Bar = 200 nm). (**c**) The AcBV-E64-G64-I64-P64-G-M-Igp64 in the supernatant was used to re-infect Sf9 cells to complete the replication of the baculovirus (24–96 h, Bar = 200 µm, Excitation = 488 nm and 522 nm). Similarly, the (**B**) indicated construction of recombinant baculovirus AcBV-Δgp64-E64-G64-I64-P64-G-M-Igp64.

Increases in cell infection rate and gene expression indicate that rBacmids encoding foreign fluorescent-protein genes (*gfp* and *mCherry*) successfully invaded Sf9 cells (Figure 9). Baculovirus particles in the supernatant were collected and analyzed by TEM and then used to re-infect normal Sf9 cells (Figure 9) to obtain the stable titer of baculoviruses.

**Figure 10:**
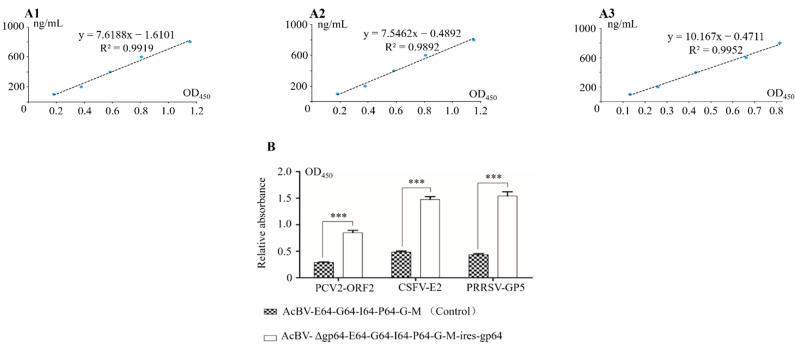
Determination the display efficiency of the antigens on the baculovirus capsule. (**A1**) ELISA standard curve of antibody (PCV2-ORF2—OD_450_). (**A2**) ELISA standard curve of antibody (PRRSV-GP5—OD_450_). (**A3**) ELISA standard curve of antibody (CSFV-E2—OD_450_). (**B**) The display efficiency of envelope antigens was determined by ELISA (***: *p* < 0.01).

To further investigate the difference in display efficiency between the two rBacmids, antigen ELISAs were performed, and the results showed that the display efficiency of PCV2 was increased by 2.93-fold, E2 by 3.04-fold, and GP5 by 3.53-fold compared with the traditional BDS (Figure 10).

### 3.4. Determination of Immunogenicity of Multiple-Vaccines Encoded by Bacmid-Δgp64

**Figure 11:**
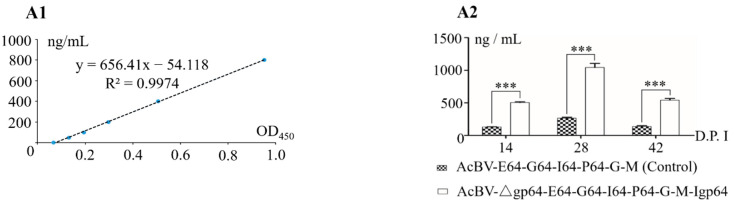

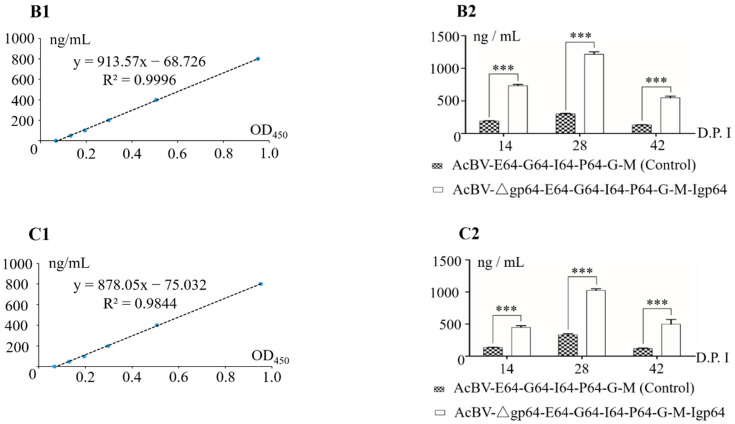
ELISA of serum-specific antibodies after immunization of mice with the recombinant baculovirus. (**A1**) ELISA standard curve of serum specific antibodies ELISA (PCV2-ORF2—OD_450_). (**A2**) The serum-specific antibodies PCV2-ORF2 were determined by ELISA at different stages of immunizing mice with AcBV-E64-G64-I64-P64-G-M and AcBV-Δgp64-E64-G64-I64-P64-G-M-Igp64 (***: *p* < 0.01). (**B1**) ELISA standard curve of serum-specific antibodies ELISA (PRRSV-GP5—OD_450_). (**B2**) The serum-specific antibodies PRRSV-GP5 were determined by ELISA at different stages of immunizing mice with AcBV-E64-G64-I64-P64-G-M and AcBV-Δgp64-E64-G64-I64-P64-G-M-Igp64 (***: *p* < 0.01). (**C1**) ELISA standard curve of serum-specificfd antibodies ELISA (CSFV-E2—OD_450_). (**C2**) The serum-specific antibodies CSFV-E2 were determined by ELISA at different stages of immunizing mice with AcBV-E64-G64-I64-P64-G-M and AcBV-Δgp64-E64-G64-I64-P64-G-M-Igp64 (***: *p* < 0.01).

A total of thirty Bal B/c mice were divided into three groups, including a control and two experimental groups (rBacmid and rBacmid-Δgp64). Mice were injected intraperitoneally with the same titer of recombinant baculoviruses AcBV-E64-G64-I64-P64-G-M (as control, virus injection titer 1 × 10^6^ PFU) and AcBV-Δgp64-E64-G64-I64-P64-G-M-iresgp64 (virus injection titer 1 × 10^6^ PFU), and the control mice were injected with the same titer of AcBV-Δgp64-p64-gfp-ires-gp64. Serum antibody ELISAs conducted on days 14, 28, and 42 after immunization showed that the expression of the three specific antibodies initially increased and then decreased. Further analyses indicated that the titers of the specific antibodies induced by recombinant Bacmid-Δgp64 were significantly higher than those obtained using traditional BDS (*p* < 0.05), with the greatest difference in titer (~4 times the control level) observed 28 days after immunization (Figure 11).

## 4. Discussion

The BDS is a eukaryotic surface display system dependent on insect cell gene expression [9]. Due to simplicity of operation and high expression and safety, the BDS is widely used for antigen presentation, antibody preparation, and receptor screening [10]. Previous research has shown that competition between GP64 and the fusion protein at the binding site on the surface of the baculovirus envelope is the primary factor affecting display efficiency [8]. The Bacmid-Δgp64 constructed in the previous study did not promote complete germination and maturation of progeny viruses due to the *gp64* gene deletion, so *ires* was used to mediate the low expression of GP64. The *ires* gene sequence effectively recruited ribosomes, resulting in complete gene transcription and protein translation. Another study showed that *ires* mediates target gene expression in HeLa and CHO cells, with 20–50% of expression due to the promoter, and this phenomenon also occurs in insect Sf9 cells [26]. The promoter p64, which mediates GP64 expression, is a typical very early promoter (12–24 h post infection); therefore, this result also used the early promoter pie1 to reduce competition and improve display efficiency [27]. In addition, the pie1 promoter exhibits high expression activity in both insect and mammalian cells, which could facilitate gene expression by recombinant baculovirus vectors in non-host cells [28,29,30].

Other reports have shown that the envelope protein GP64 primarily mediates baculovirus cell-invasion processes, including electrostatic adsorption, membrane protein recognition, and pinocytosis [31,32,33]. Although the rBacmid-Δgp64-ires-gp64 constructed in this study exhibited improved display efficiency, it also reduced the titer and infection rate of the recombinant baculovirus. In a previous study, we demonstrated direct invasion of *E. coli* into insect Sf9 and BmN cells via INVASIN [8]. In the present study, we also employed envelope display of protein INVASIN and VSVG to significantly improve the invasion efficiency of recombinant rBacmid-Δgp64-ires-gp64 in mammalian cells (*p* < 0.05). Further analyses indicated that VSVG is cytotoxic, which significantly promotes the fusion of infected Sf9 cells [34,35]. It has been reported that the *Red-gam* technology was used to delete the N-terminal ectodomain to prevent cell fusion, but this deletion abolished the VSVG function of improving the efficiency of baculovirus transduction [36,37].

As baculoviruses can stimulate the immune system, they are excellent recombinant virus vectors due to the vaccine adjuvant effect [38,39,40]. However, when a baculovirus vector enters a vertebrate animal, it is quickly recognized and inactivated by the complement system in serum or other body fluids [41]. Therefore, avoiding complement inactivation and prolonging the immunization duration are critical issues that have thus far limited the application of baculovirus vectors. Previous studies have shown that recombinant virus particles displaying Cobra venom factors on the envelope can bind to the alternative factor of complement C3, thus improving the efficiency of transduction and expression of viral vector genes in hepatocytes [42]. By co-displaying the C5α receptor and compstatinis, the recombinant baculovirus also significantly enhanced the antigen presentation efficiency, immune response, and cytoinflammatory factor expression of recombinant viral vectors [43]. In addition, DAF, factor H protein, C4b-binding protein, and cell membrane cofactor can also improve the anti-inactivation ability of virus particles [44,45]. Therefore, stable and efficient co-display of multiple target proteins and improvements in the complement-inactivation ability of the recombinant baculovirus will set a new stage for BDS in the field of multiple-vaccine development.

## 5. Conclusions

We have provided evidence that the BDS is a useful virus vector for genetic engineering of vaccines. We developed a recombinant baculovirus vector exhibiting higher display efficiency and higher virus titer compared with previous systems in addition to improved efficiency of baculovirus transduction into mammalian cells. Compared with traditional BDSs, the titers of specific antibodies against the three displayed antigens (PCV2-ORF2, PRRSV-GP5, and CSFV-E2) could be increased by approximately 4-fold, thereby maximizing the immunological advantages of recombinant baculovirus vector vaccines.

## Figures and Tables

**Table 1:** Primers used to amplify the target gene.

Label	Primer Sequences	Genes
*Rluc*	F: 5′-tctagaatgatgtacatagaagatt-3′ (*Xba* I) R: 5′-ctgcagttacacttgatacatgc-3′ (*Pst* I)	*Renilla*-luciferase
*Fluc*	F: 5′-cccgggatggaagacgccaaaaacataaagaa-3′ (*Xma* I) R: 5′-ctcgagttacacggcgatctttccgcccttctt-3′ (*Xho* I)	*Firefly*-luciferas
*p10*	F: 5′-actagtatacggacctttaattcaacccaac-3′ (*Spe* I) R: 5′-cccgggtgatcaagtcttcgtcgagtgatt-3′ (*Xma* I)	promoter of baculovirus
*pvp39*	F: 5′-actagttcgcgagttttgcagcgtctctgaa-3′ (*Spe* I) R: 5′-cccgggggatccttgttgccgttataaatatg-3′ (*Xma* I)	promoter of baculovirus
*pcmv*	F: 5′-actagtgttgacattgattattgtctagtta-3′(*Spe* I) R: 5′-cccgggtctgcttatatagacctcccaccgta-3′ (*Xma* I)	promoter of baculovirus
*pie1*	F: 5′-actagttcaattttatgtggctaatggagaat-3′ (*Spe* I) R: 5′-cccgggcttgagtggagagagagagctagtta-3′ (*Xma* I)	promoter of white spot syndrome virus
*psv40*	F: 5′-actagtattcaaatatgtatccgctcatgagac-3′ (*Spe* I) R: 5′-cccgggcctccaaaaaagcctcctcactactt-3′ (*Xma* I)	promoter of Simian vacuolating virus 40
*vsvg*	F: 5′-cccgggatggtaagcgctattgttttatatgtgcttttggcggcggcggcgcattctgcctttgc ggcgggatctaagttcaccatagttt-3′ (*Xma* I) R: 5′-ctcgagttactttccaagtcggttcatctcta-3′ (*Xho* I)	envelope gene of vesicular stomatitis virus
*invasin*	F: 5′-cccgggatggtaagcgctattgttttatatgtgcttttggcggcggcggcgcattctgcctttgc ggcgatggataacgatgttgctaataata-3′ (*Xma* I) R: 5′-ctcgagtattgccagcgcacagagcgggaacg-3′ (*Xho* I)	infection gene of *Yersinia pseudotuberculosis*
*E2*	F: 5′-cccgggatggtaagcgctattgttttatatgtgcttttggcggcggcggcgcattctgcctttgc ggcgcggctagcctgcaaggaaga-3′ (*Xma* Ⅰ) R: 5′-ctcgaggtagaatagatcttcattttccact-3′ (*Xho* Ⅰ)	envelope gene of classical swine fever virus
*gp5*	F: 5′-ggatccatggtaagcgctattgttttatatgtgcttttggcggcggcggcgcattctgcctttgc ggcgttggggaagtgcttgaccgcgt-3′ (*Bam*H Ⅰ) R: 5′-gagctcgagacgaccccattgttccgctgaaa-3′ (*Sac* I)	envelope gene of porcine reproductive and respiratory syndrome virus
*pcv2*	F: 5′-ggatccatggtaagcgctattgttttatatgtgcttttggcggcggcggcgcattctgcctttgc ggcgacgtatccaaggaggcgttacc-3′ (*Bam*H I) R: 5′-gagctcttcattaagggttaagttgggggtct-3′ (*Sac* Ⅰ)	envelope gene of porcine circovirus type 2
*64ORF*	F: 5′-gagctcctcgaggagcactgcaacgcgcaaatgaag-3′ (*Sac* I + *Xho* I) R: 5′-ctgcaggcatgcttaatattgtctattacggtttctaa-3′ (*Pst* I + *Sph* I)	baculoviral envelope gene

The 5′ ends of primers were designed to create restriction enzyme sites (underlined), respectively. The shaded area is GP64 protein signal peptide (sp).

## Data Availability

Data are contained within the article.
